# VCP regulates early tau seed amplification via specific cofactors

**DOI:** 10.21203/rs.3.rs-4307848/v1

**Published:** 2024-05-22

**Authors:** Sushobhna Batra, Jaime III Vaquer-Alicea, Clarissa Valdez, Skyler P Taylor, Victor A Manon, Anthony R Vega, Omar M Kashmer, Sourav Kolay, Andrew Lemoff, Nigel J Cairns, Charles L White, Marc I Diamond

**Affiliations:** UT Southwestern: The University of Texas Southwestern Medical Center; UT Southwestern: The University of Texas Southwestern Medical Center; UT Southwestern: The University of Texas Southwestern Medical Center; UT Southwestern: The University of Texas Southwestern Medical Center; UT Southwestern: The University of Texas Southwestern Medical Center; UT Southwestern: The University of Texas Southwestern Medical Center; UT Southwestern: The University of Texas Southwestern Medical Center; UT Southwestern: The University of Texas Southwestern Medical Center; UT Southwestern: The University of Texas Southwestern Medical Center; University of Exeter Faculty of Health and Life Sciences; UT Southwestern: The University of Texas Southwestern Medical Center; UT Southwestern: The University of Texas Southwestern Medical Center

**Keywords:** Tau, APEX2, VCP, p97, Cofactors, Disaggregase, Seeding

## Abstract

**Background:**

Neurodegenerative tauopathies may progress based on seeding by pathological tau assemblies, whereby an aggregate is released from one cell, gains entry to an adjacent or connected cell, and serves as a specific template for its own replication in the cytoplasm. In vitro seeding reactions typically take days, yet seeding into the complex cytoplasmic milieu happens within hours, implicating a machinery with unknown players that controls this process in the acute phase.

**Methods:**

We used proximity labeling to identify factors that control seed amplification within 5h of seed exposure. We fused split-APEX2 to the C-terminus of tau repeat domain (RD) to reconstitute peroxidase activity 5h after seeded intracellular tau aggregation. Valosin containing protein (VCP/p97) was the top hit. VCP harbors dominant mutations that underlie two neurodegenerative diseases, multisystem proteinopathy and vacuolar tauopathy, but its mechanistic role is unclear. We used immortalized cells and human neurons to study the effects of VCP on tau seeding. We exposed cells to fibrils or brain homogenates in cell culture media and measured effects on uptake and induction of intracellular tau aggregation following various genetic and chemical manipulations of VCP.

**Results:**

VCP knockdown reduced tau seeding. Chemical inhibitors had opposing effects on aggregation in HEK293T tau biosensor cells and human neurons alike: ML-240 increased seeding efficiency, whereas NMS-873 decreased it. The inhibitors were effective only when administered within 8h of seed exposure, indicating a role for VCP early in seed processing. We screened 30 VCP co-factors in HEK293T biosensor cells by genetic knockout or knockdown. Reduction of ATXN3, NSFL1C, UBE4B, NGLY1, and OTUB1 decreased tau seeding, as did NPLOC4, which also uniquely increased soluble tau levels. By contrast, reduction of FAF2 increased tau seeding.

**Conclusions:**

Divergent effects on tau seeding of chemical inhibitors and cofactor reduction indicate that VCP regulates this process. This is consistent with a dedicated cytoplasmic processing complex based on VCP that directs seeds acutely towards degradation vs. amplification.

## Introduction

Neurodegenerative tauopathies include Alzheimer’s and related disorders that are caused by intracellular accumulation of pathological tau assemblies (1). In each disease, pathology progresses predictably, possibly via connected neural networks (2–5). Experimental and observational evidence suggests that this occurs by release of tau aggregates, followed by their entry into a second order cell. The assembly then serves as a precise template for its own replication, a process termed “seeding,” which is easily replicated in simple cell models, cultured neurons, and mouse brain (6–11).

*In vitro* amplification of tau assemblies from fibrillar seeds typically takes several days, even under optimized conditions (12,13), whereas in cells this occurs more quickly, sometimes within hours, and in certain cases will faithfully reproduce specific assembly structures (9). Interestingly, no seed amplification assay *in vitro* has yet achieved the fidelity of structural replication that occurs in cells, consistent with a role for co-factors. We originally found that tau aggregates bind heparan sulfate proteoglycans (HSPGs) on the surface and are taken up via macropinocytosis (14). Most tau then traffics to the endolysosomal system where it is degraded by lysosomal proteases (15). By contrast, a small fraction of seeding activity steadily enters the cytoplasm with clearance by the proteasome (15). Seed amplification occurs widely throughout the cytoplasm, and is not necessarily associated with the original large aggregates (15). These observations, and others, have led us to speculate that tau seeding is regulated by an intracellular “machinery” that brings an assembly into contact with free tau monomer for amplification. Several proteomics screens from our lab and others have identified proteins associated with pre-existing intracellular tau aggregates (16–20), including valosin containing protein (VCP/p97) (19,20), but we still do not understand the factors involved early in seed amplification, and conflicting reports suggest that VCP could increase (20) or decrease seeding by exogenous amyloids (21,22). In this study, we used proximity labeling 5h after aggregate exposure to identify VCP as a top hit, and have characterized its regulatory role at the earliest stages of tau seed amplification.

## Results

### Proximity labeling of nascent tau aggregates identifies VCP

To identify proteins nearby tau as it initiated aggregation, we exploited split-APEX2 (sAPEX2), which renders the enzyme inactive until holoenzyme reconstitution (23). We fused tau repeat domain (RD) containing the disease-associated P301S mutation to APEX2 fragments (AP: aa1–200; EX: aa201–250) each followed by an IRES sequence fused to either blue fluorescent protein or mCherry to confirm expression of both constructs. As a negative control, we used tau containing two proline substitutions (I277P / I308P) that prevented formation of beta-sheet structures (24,25). We also used tau RD WT to compare to P301S and dual proline mutants. Unfused sAPEX2 expression alone controlled for background enrichment of any non-specific proteins.

**Figure 1:**
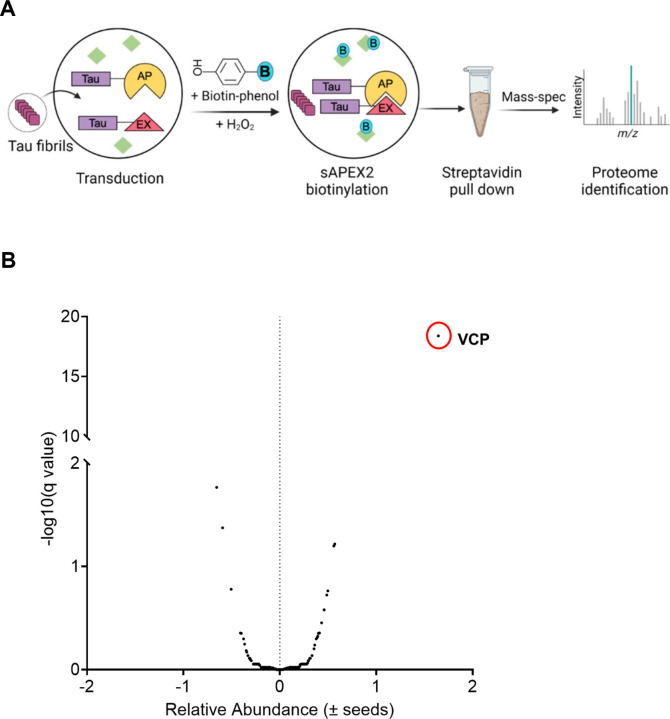
VCP identified by proximity labeling from tau aggregation. **(A)**Schematic of the TMT-MS study performed for proteomics. **(B)** VCP was identified as the most significant hit enriched in the early tau aggregation proteome. Unpaired t- test, P value < 0.000001.

We induced tau RD-AP/EX aggregation by Lipofectamine-mediated transduction of cells with full-length (FL), wild-type (WT) tau fibrils. The earliest detectable biotinylation occurred 5h after induction (Supplemental Fig. 1A). After transduction of cells with tau fibrils, we thus waited 5h before treating with biotin-phenol (BP) and H_2_O_2_. We then lysed the cells and used streptavidin beads to purify biotinylated proteins, which we identified using tandem mass-tag mass spectrometry (TMT-MS), pooling data from three independent experiments (Fig. 1A). VCP/p97 was the most significantly enriched hit (Fig. 1B).

VCP (known as Cdc48 in yeast and Ter94 in fruit flies) is a AAA+ ATPase with two ATPase domains, D1 and D2. Its N-terminus binds specific cofactor/adaptor proteins that govern its diverse cellular activities (26,27). A dominant mutation in VCP causes vacuolar tauopathy (VT), a neurodegenerative syndrome (21), and other mutations cause multisystem proteinopathy (MSP), with protein aggregation and degeneration in brain, bone, and muscle (28,29). Recently, work from our lab and in an independent collaboration with the Hipp and Hartl laboratories identified VCP associated with insoluble tau aggregates in cells that stably propagate inclusions (19,20). It has been unclear how VCP might regulate intracellular seeding to promote (20) or prevent it (21,22).

### VCP differentially regulates tau seeding

**Figure 2:**
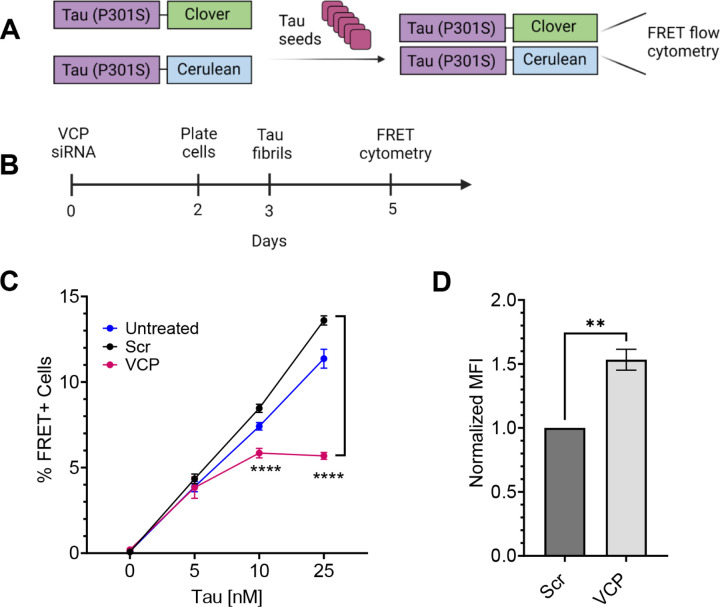
Reduction of VCP inhibits tau seeding. **(A)** The tau biosensor seeding assay is based on exposure of cells to exogenous tau seeds which trigger intracellular aggregation of tau RD (P301S)-cerulean/cloverthat is detected by FRET. **(B)** Timeline of siRNA treatment to knockdown (KD) VCP in biosensors. **(C)** KD of VCP reduced tau seeding. Error bars represent S.D. Graph represents n=3 independent experiments. P value **** < 0.0001; One-Way ANOVA with a 95% confidence interval. **(D)** VCP KD increased uptake of tau fibrils labeled with AF-647, measured by flow cytometry. Error bars represent S.E.M (n=3). P value ** 0.0029; Unpaired t-test with a 95% confidence interval.

To quantify tau aggregation, we used v2L biosensor cells that overexpress tau RD (P301S) tagged with mClover3 and mCerulean3, termed tau RD (P301S)-Clover/Ruby (Fig. 2A) (8). In contrast to the original APEX2 screen, hereafter we added recombinant tau fibrils to the media in the absence of a transfection reagent to enable HSPG-mediated macropinocytosis (7,14,30) and cytoplasmic seeding (6,31,15), which we quantified using FRET flow cytometry (7). We genetically and pharmacologically modulated VCP activity in the biosensors to test its effects on tau seeding. Knockout (KO) of VCP is lethal, so we used siRNA-mediated knockdown (KD) (Fig. 2B), verified by western blot (Supplemental Fig. 2A). VCP KD reduced tau seeding (Fig. 2C). We also observed increased basal fluorescence of the biosensor cells by microscopy (Supplemental Fig. 2B) and flow cytometry (Supplemental Fig. 2C). This was consistent with VCP-mediated degradation of tau monomer and made the reduction of overall seeding efficiency more remarkable (Fig. 2C, Supplemental Fig. 2D).

VCP can affect vesicular trafficking (32), so to rule out reduction of tau uptake as a cause of diminished seeding, we treated the biosensors with AF-647 (Alexa fluor-647) labeled recombinant tau fibrils for 4h, followed by trypsin digestion to degrade extracellular fibrils. We measured uptake via flow cytometry as per standard protocols (14,30). VCP KD slightly increased tau uptake (Fig. 2D), ruling out diminished endocytosis as the cause of the reduced seeding.

**Figure 3:**
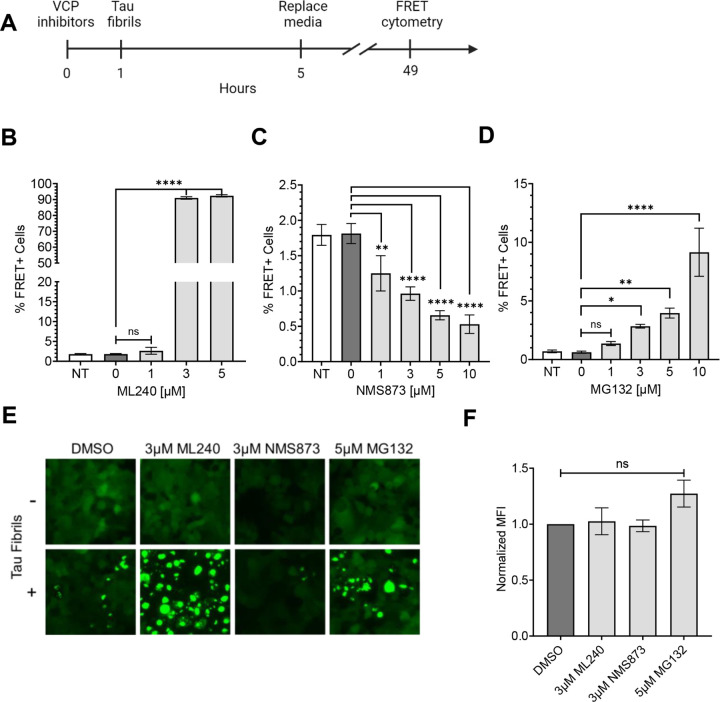
Acute exposure of inhibitors differentially impacts tau aggregation. **(A)** Timeline of experiment: 1h exposure of tau biosensor cells to inhibitors, followed by 4h of 25nM tau, before washout. **(B)** ML-240 dose-dependently increased tau seeding. P values: ns = 0.4353, **** < 0.0001**(C)** NMS-873 dose-dependently decreased tau seeding. Error bars represent S.D. Representative data of n=3 independent experiments. P values: ** 0.0030, **** < 0.0001 **(D)** Proteasome inhibitor MG132 increased tau seeding. P values: ns = 0.8492, * 0.0415, ** 0.0024, **** < 0.0001 **(E)** Fluorescence microscopy confirmed the effects of VCP and proteasome inhibition on tau seeding. **(F)** Compound treatment did not change tau-AF-647 uptake as measured by flow cytometry. Error bars represent S.E.M (n=3). P values: ns = 0.9960, 0.9992, 0.1742, in order of bars on the graph. One-Way ANOVA with a 95% confidence interval.

Chronic VCP KD reduced cell proliferation over time and induced toxicity (Supplemental Fig. 2B,C). Thus, we temporarily inhibited VCP using two distinct inhibitors, ML-240 and NMS-873 (Fig. 3A). ML-240 competitively blocks ATP binding at D2, whereas NMS-873 allosterically inhibits VCP by binding to the linker between the D1 and D2 domains (33–35). We pre-treated the cells with inhibitors for 1h, then incubated them with tau fibrils for 4h, followed by washout and measurement of seeding at 48h. We observed opposing effects: ML-240 increased tau aggregation from approximately 2% to ~90% (Fig. 3B, E) and speeded aggregation kinetics (Supplemental Fig. 3A). By contrast, NMS-873, reduced tau aggregation by ~50% (Fig. 3C,E). Because VCP regulates protein degradation via the proteasome (33,36,37), we repeated the study with MG132. This increased tau seeding from ~1% to ~10% at 48h (Fig. 3D, E). This agreed with our prior observation that the proteasome mediates cytoplasmic clearance of seeds (15). None of the compounds altered tau uptake (Fig. 3F).

**Figure 4:**
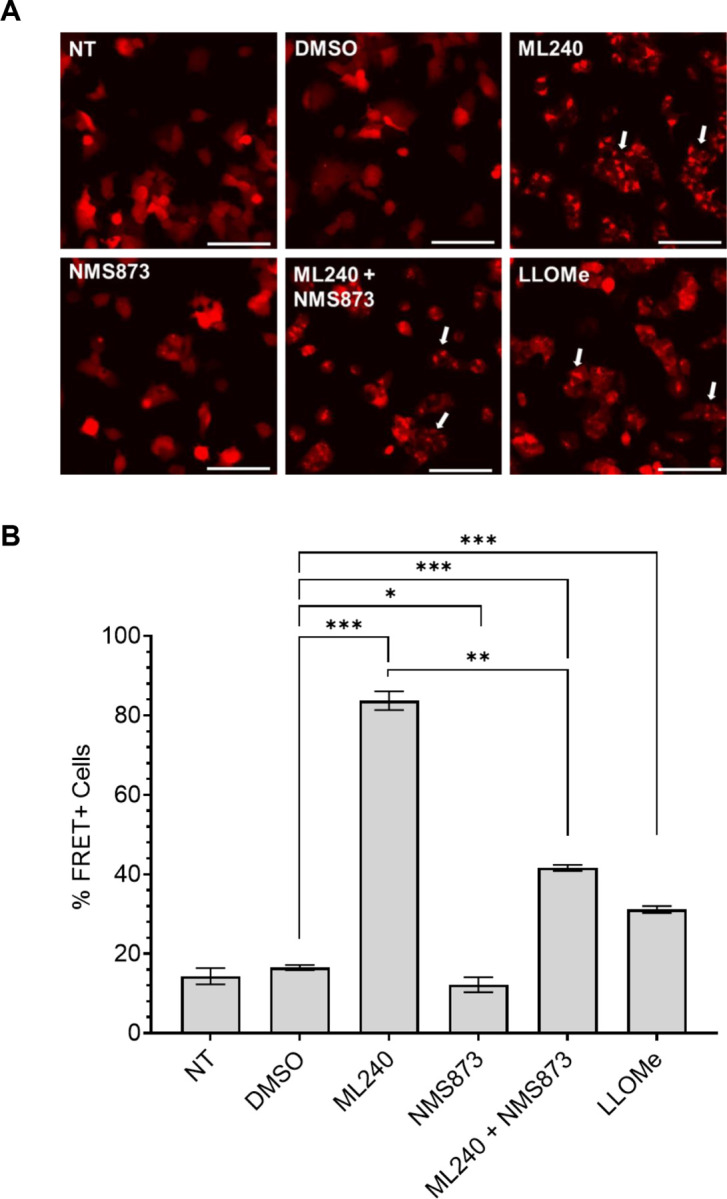
ML-240 induces Gal3 puncta formation. v2L biosensors overexpressing galectin-3-mRuby3 were treated with different compounds for 1h prior to addition of tau fibrils and media replacement after 4h. **(A)** Both ML-240 (3μM) and LLOMe (1mM) induced Gal3 puncta with no effects of NMS-873 (3μM). Co-treatment of ML-240 and NMS-873 also induced Gal3 puncta. Representative images of n=3 independent experiments taken before media replacement (5h of compound alone incubation). **(B)** ML-240 increased tau seeding ~5x; LLOMe induced seeding by ~2x. NMS-873 suppressed the tau seeding enhanced by ML-240. Representative data of n=3 independent experiments. Error bars represent S.D. P values: **** 0.005, * 0.0331, *** 0.0007, ** 0.0017, *** 0.0001 in order of the bars on the graph. One-Way ANOVA with a 95% confidence interval. Scale bar = 100mm.

The remarkable increase in tau seeding with ML-240 prompted us to check if its effects were mediated via vesicle rupture, thereby increasing the amount of tau seeds available in the cytoplasm for amplification. We utilized v2L biosensors overexpressing galectin-3 tagged to mRuby3 fluorophore (v2L-Gal3) to visualize Gal3 puncta formation. We also overexpressed the same construct in U2OS cells for imaging. We used the lysosomotropic reagent L-Leucyl-L-leucine methyl ester (LLOMe) as a positive control to induce endolysosome rupture (38–40). Brief exposure of both v2L-Gal3 and U2OS cells with ML-240 and LLOMe induced Gal3 puncta formation, indicating vesicle rupture (Fig. 4A; Supplemental Fig. 4A), however ML-240 enhanced seeding far more than LLOMe (Fig. 4B). NMS-873 did not induce Gal3 puncta (Fig. 4A; Supplemental Fig. 4A). When we co-treated cells with ML-240 and NMS-873, Gal3 puncta formed (Fig. 4A; Supplemental Fig. 4A) but seeding was relatively attenuated, indicating that NMS-873 likely inhibited a process downstream of vesicle rupture (Fig. 4B).

**Figure 5:**
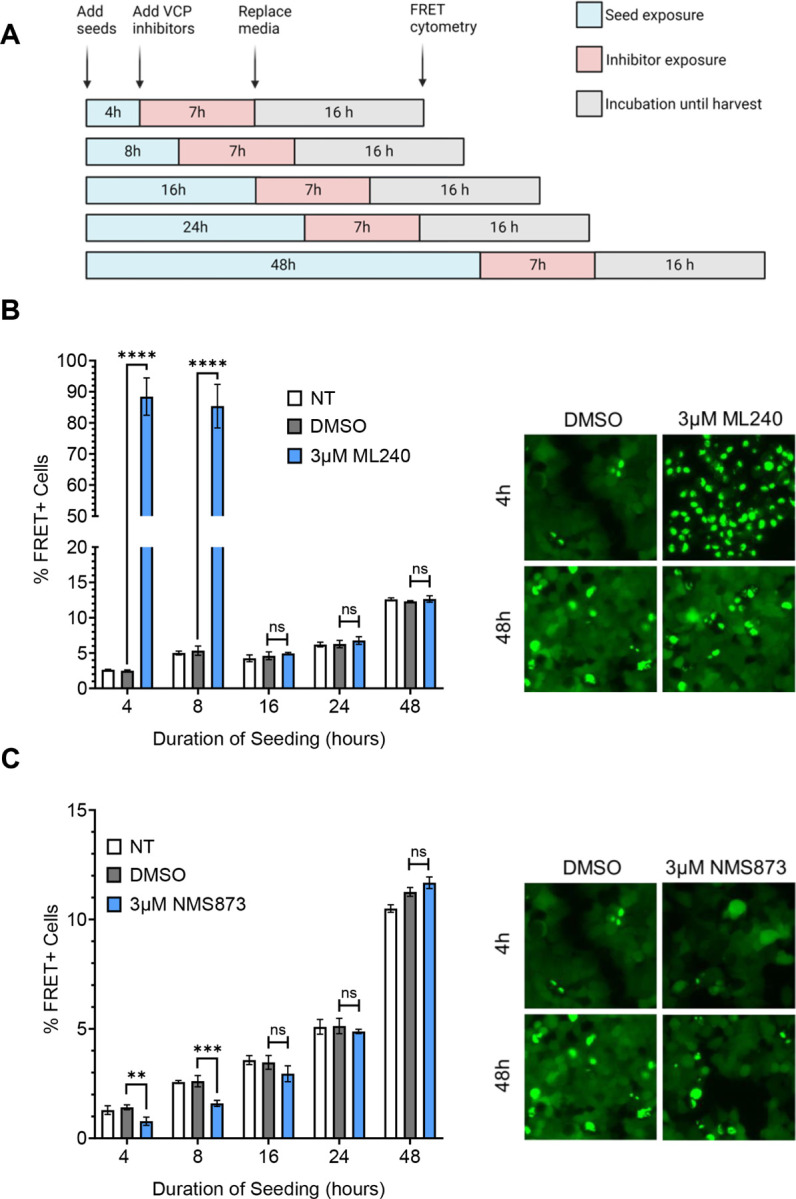
VCP inhibition impacts tau seedingearly in the process. **(A)** Timeline of compound and tau treatments at different time points in the seeding process. **(B)** ML-240 increased tau aggregation ~16 to 25-fold, but only when administered <8h after seed exposure. Representative images (20x magnification) are shown in the right panel. P values: **** < 0.0001, ns (16hr) = 0.5892, ns (24hr) = 0.4340, ns (48hr) = 0.3569 **(C)** NMS-873 decreased tau seeding by ~50%, but only when administered <8h after seed exposure. Representative images (20x magnification) are shown in the right panel. P values: ** 0.0039, *** 0.0004, ns (16hr) = 0.0788, ns (24hr) = 0.8695, ns (48hr) = 0.0547. Error bars represent S.D. Representative data of n=3 independent experiments. One-Way ANOVA with a 95% confidence interval.

VCP regulates protein degradation, among other functions, and thus could impact seeding through clearance of aggregates over time. To resolve this issue, we added inhibitors for 7h beginning at different time points after initial seed exposure (Fig. 5A). The inhibitors changed tau seeding only when administered within the first 8h, and we observed no effect on seeding after that timepoint (Fig. 5B, C; Supplemental Fig. 5A). These results indicated that VCP regulates aggregation early in the seeding process.

**Figure 6:**
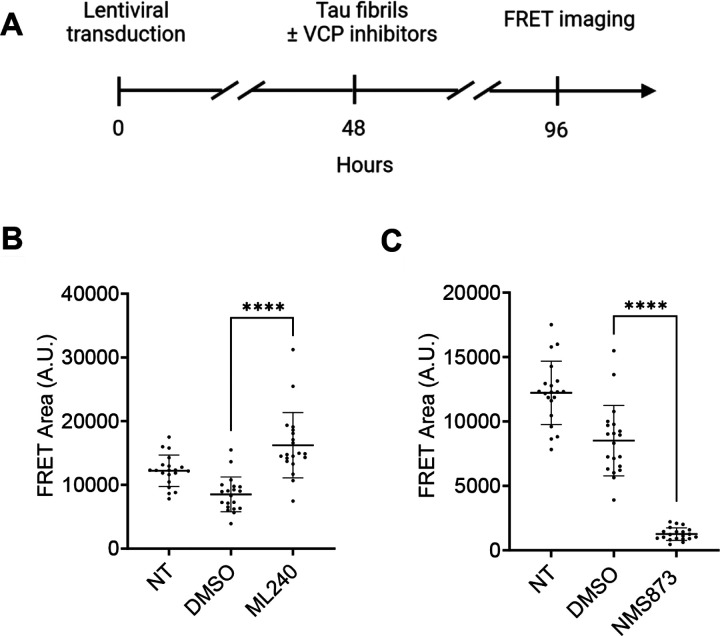
VCP inhibitors differentially impact tau seeding in human neurons. **(A)** Differentiated iPSC-derived human neurons were transduced with tau RD(P301S)-clover/ruby lentivirus for 48h followed by seeding in the presence or absence of 100nM VCP inhibitors. **(B)** ML-240 enhanced seeded tau aggregation in neuronal biosensors whereas **(C)**NMS-873 suppressed tau seeding. Error bars represent S.D. Representative data for n=3 independent experiments. Each dot represents an image taken per condition, with 4 different points captured per well, for a total of 5 wells per condition. P value: **** < 0.0001; Paired t-test with a95% confidence interval.

To test the effects of VCP inhibitors in a more physiologically relevant system, we measured tau seeding in iPSC-derived human neurons treated with ML-240 and NMS-873. We first transduced differentiated neurons with lentivirus to express tau RD (P301S)-Clover/Ruby biosensor proteins. After 48h, we treated cells with ML-240 or NMS-873 at 100nM along with tau fibrils for an additional 48h and measured FRET (Fig. 6A). We also measured seeding in human neurons treated with the inhibitors for 4h (ML-240 at 1μM and NMS-873 at 100nM) prior to washout and culture for 48h (Supplemental Fig. 6A). We quantified seeding by high content FRET microscopy (Image Xpress). ML-240 increased and NMS-873 decreased tau seeding (Fig. 6B,C; Supplemental Fig. 6B,C).

### ML-240 increases AD and CBD brain lysate seeding

**Figure 7:**
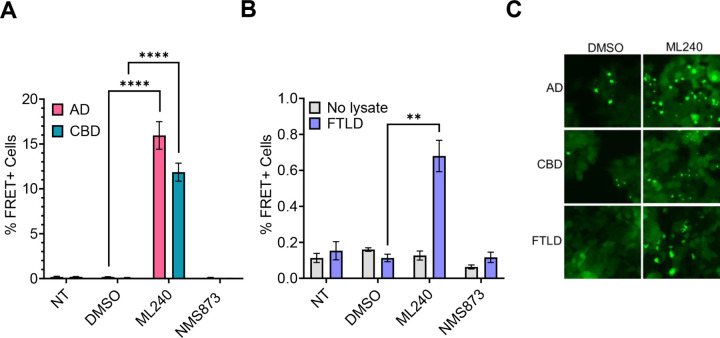
ML-240 enhances seeding by human brain lysates. **(A)** ML-240 increased seeding by AD and CBD brain samples into tau biosensors. **(B)** ML-240 increased seeding by FTLD type A brain lysate onto TDP-43 biosensors. Error bars represent S.D. Representative data for n =3 independent experiments. **(C)** Representative microscopy images showing effects of ML-240 on tauopathy lysates and FTLD seeding as observed under the YFP channel. Error bars represent S.D. Representative data of n=3 independent experiments. P value: **** < 0.0001, ** 0.0088; One-Way ANOVA with a 95% confidence interval.

To test the effects of VCP inhibitors on a physiological tau seed source, we tested tauopathy brain lysates from AD (Alzheimer’s disease) and CBD (corticobasal degeneration) patients. We treated biosensor cells with ML-240 and NMS-873 as described above, after which they were exposed to AD and CBD patient brain lysates without a transduction reagent. ML-240 increased AD and CBD seeding by ~10x (Fig. 7A, C). Since the lysates induced very low seeding in the absence of a transduction reagent, we could not test the effects of NMS-873.

### ML-240 increases TDP-43 brain lysate seeding

To test whether VCP regulated seeding by another amyloid, we evaluated the effects of the inhibitors on biosensors expressing WT TDP-43 (a.a. 275–414) fused to mClover3 or mRuby3 fluorescent proteins. ML-240 enhanced FTLD-TDP Type A brain lysate seeding on TDP-43 biosensors (Fig. 7B, C). Since the lysate induced no seeding in the absence of a transduction reagent, we could not test the effects of NMS-873.

### VCP co-factors regulate tau aggregation

Multiple co-factors generate specificity for VCP’s myriad cellular functions (26,41,42). To identify those which regulate seeding in v2L tau biosensors, we individually knocked out or reduced the expression of 30 cofactors known to be expressed in HEK293T cells (43) (Table 1).

For CRISPR/Cas9 KO, we used four gRNAs per gene from the Brunello library (44), compared to 4 non-targeting guides (NTG) combined as a negative control. For genes that were toxic upon KO, we used siRNA-mediated KD, with scrambled (Scr) siRNA as a negative control. KO or KD of most cofactors did not change tau seeding (Supplemental Fig. 8A). RPS27A was the only cofactor for which both KD and KO were lethal and thus we could not determine its effects on seeding.

**Figure 8:**
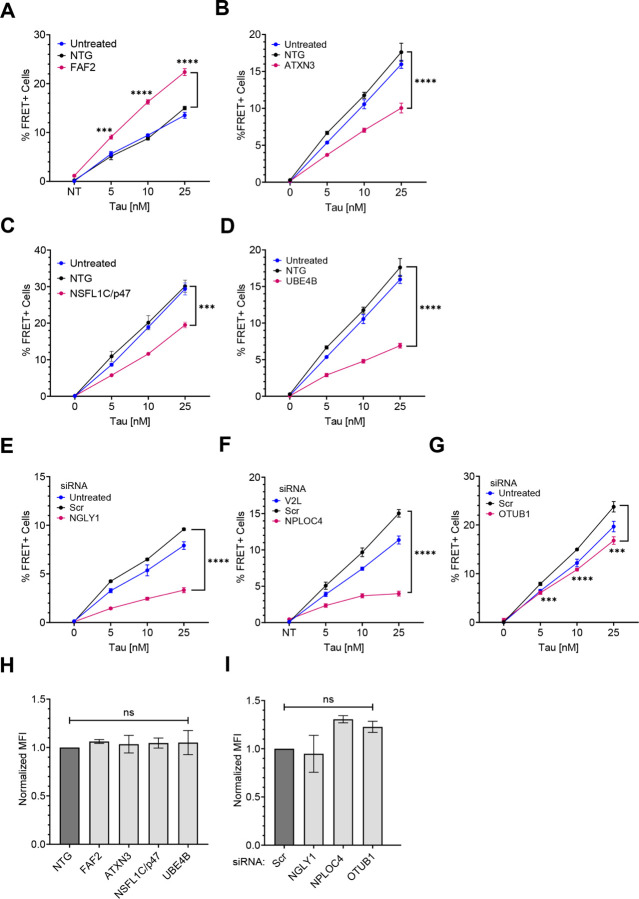
VCP cofactors differentially regulate tau seeding. VCP cofactors were either knocked out via CRISPR/Cas9 (**A-D**) or knocked down via siRNA (**E-G**) in v2L biosensors prior to exposure to increasing amounts of tau fibrils. **(A)** Knockout of FAF2 increased tau seeding whereas knockout of **(B)** ATXN3, **(C)** NSFL1C, and **(D)** UBE4B reduced tau seeding. P values: FAF2 (*** 0.0001, **** < 0.0001); ATXN3 (**** <0.0001); NSFL1C (*** 0.0002); UBE4B (**** < 0.0001). **(E)** Knockdown of NGLY1, **(F)**NPLOC4, and **(G)** OTUB1, decreased tau seeding. P values: NGLY1(**** < 0.0001); NPLOC4 (**** < 0.0001); OTUB1 (*** 0.0004, **** < 0.0001, *** 0.0001). Graphs are representative of n= 3 independent experiments. Error bars represent S.D. **(H)** Cofactor KO did not affect tau uptake. P values: ns = 0.9819, 0.9988, 0.9956, 0.9928, in order of bars on the graph. **(I)** Cofactor KD did not affect tau uptake. P values: ns = 0.9795, 0.1856, 0.3928, in order of bars on the graph. Graphs are representative of n= 3 independent experiments. Error bars represent S.E.M. One-Way ANOVA with a 95% confidence interval.

Knockout of UBXN6 increased tau seeding but the effect was most pronounced at higher tau concentrations (Supplemental Fig. 8B). KO of FAF2 alone clearly increased tau seeding (Fig. 8A), and even induced spontaneous aggregation in the biosensors in the absence of any exogenous tau fibrils (Supplemental Fig. 8C,D). In contrast, KO of the deubiquitinase ATXN3, and the E3/4 ligase UBE4B, suppressed seeded tau aggregation (Fig. 8B,D). KO of NSFL1C also reduced seeding (Fig. 8C). We validated each effective KO by western blot (Supplemental Fig. 8E). siRNA identified three cofactors whose KD decreased seeding: NGLY1, NPLOC4, and OTUB1 (Fig. 8E,F,G)). Remarkably, KD of NPLOC4 increased tau levels in the biosensors (Supplemental Fig. 8F,G) yet reduced seeding (Supplemental Fig. 8F,H). NPLOC4 KD increased inclusion size, whereas NGLY1 KD reduced fluorescence and created smaller puncta (Supplemental Fig. 8F). We validated each effective KD by western blot (Supplemental Fig. 8I). No cofactor KO or KD changed tau uptake (Fig. 8H,I).

## Discussion

It is unknown whether a cellular regulatory machinery might control seeding by tau, especially within the first hours of cell entry. To identify factors that participate in tau seed amplification within 5h of exposure, we used proximity labeling to identify VCP, which has previously been genetically and biochemically linked to chronic tau aggregation (19–21), and to inhibition of a-synuclein and TDP-43 seeding (22). Chemical manipulations of VCP up- and down- regulated tau seeding in immortalized cells and human neurons. Interestingly, these effects only manifested if inhibitors were applied within 8h of seed exposure. We observed these effects also with brain-derived tau and TDP-43 seeds, along with another amyloid substrate, recombinant α-synuclein fibrils (Supplemental Fig. 7A). We identified select VCP cofactors that participate in this differential regulation, suggesting that a complex within the cell processes incoming tau seeds, either to decrease or increase their replication efficiency.

### VCP processes seeds to determine their fate

Our prior work defined a trafficking pathway for tau seeds that delivers them to the cytoplasm, where they are cleared by the proteasome (15), and in this study we found acute proteasome inhibition increased seeding. Ordinarily, seeding efficiency from aggregates that enter the cell via endocytosis is relatively low, consistent with robust clearance mechanisms (15). However, we observed a dramatic increase in seeding efficiency for recombinant and patient-derived seeds in the presence of ML-240, a VCP inhibitor that targets the D2 ATPase. Conversely, NMS-873, an allosteric inhibitor, reduced tau seeding by ~50%, as did knockdown of VCP. We observed a seemingly paradoxical effect in the context of knockdown of VCP and NPLOC4: an increased tau steady state and reduced seeding. This suggests a core function of VCP in up- and down-regulating tau seeding that is linked to, but functionally independent of tau degradation.

**Figure 9:**
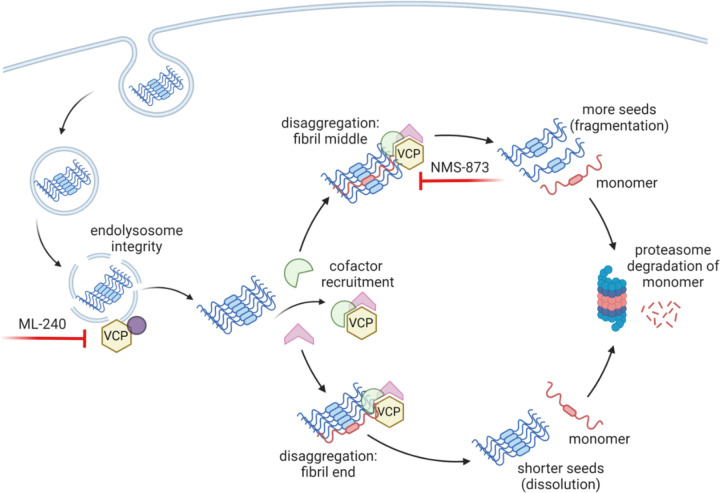
VCP regulation of tau seeding. VCP regulates endolysosome integrity, which governs the amount of tau seeds escaping into the cytoplasm. VCP then acts on tau seeds that enter the cytoplasm either to promote degradation or amplification. Disaggregase activity of VCP removes monomer for degradation. This might occur at the end of filaments, which would decrease seeding, or from within, which could fragment fibrils and promote seeding. Chemical inhibitors and cofactors bias the process towards different processing paths.

Our proposed model (Fig. 9) represents a VCP-mediated balance of forces that could decrease or increase seeding, and is based largely on studies of the yeast prion disaggregase, Hsp104, which regulates prion propagation and dissolution (45–47). Synthesizing our recent data with prior work on VCP and Hsp104, we hypothesize that VCP could regulate seeding in two ways. First, by influencing membrane repair, it might affect seed escape through the endolysosomal membranes. Second, depending on where it extracts tau monomer from the amyloid assembly (middle vs. end), it could progressively reduce fibril length (to reduce seeding) or fragment fibrils (to increase seeding) (Fig. 9).

Others have proposed slightly different models for VCP’s effects on amyloids. Darwich et al. suggested that the vacuolar tauopathy mutation reduces VCP disaggregase activity (21), and hence clearance of existing tau aggregates; whereas Zhu et al. proposed that VCP-mediated surveillance of damaged vesicles and lysophagy governed suppressed a-synuclein aggregation, although this was based on cation-mediated amyloid delivery to biosensors that bypassed standard endocytosis (22). Endolysosome rupture has been observed to facilitate enhanced tau seeding as mediated by ESCRT III machinery proteins such as CHMP6 (48), and recent work also indicates that lysosomal micro-rupture (without production of Gal3-postive puncta) allows exogenous seeds to access the cytoplasm (49). It is now well established that VCP, along with specific cofactors, regulates the endolysosomal damage response (ELDR) pathway which is altered by VCP disease-associated mutations (50–53). Taken together with findings here and previously reported (15), it seems likely that VCP plays a significant role in regulation of early seed access to the cytoplasm based on facilitation of endolysosomal leakage. NMS-873 counteracted the ML-240-mediated increase in tau seeding consistent with an effect on fibril fragmentation downstream of cytoplasmic entry. The model makes specific predictions that will require further testing to identify the precise mechanisms by which VCP prevents (21,22,54) or promotes (20) seeding by tau and other amyloid proteins.

### VCP functions early in the seeding process

We designed the proximity labeling to identify proteins adjacent to newly formed tau aggregates at the earliest possible time point, and identified VCP as the single, most reliable hit. The low number of identified proteins may be because APEX2 holoenzyme reconstitution restricted the labeling efficiency. Our lab and others have clearly observed VCP association with mature aggregates (19,20), implying additional roles in chronic protein quality control. However, the restriction of VCP’s effects on seeding to early in the process indicate a critical role of VCP in processing of tau seeds as they first enter the cytoplasm. We note conflicting reports about how seeds might exit the endolysosomal system (15,31,49). Regardless, we have previously found that tau seeds in the cytoplasm are cleared by the proteasome, and not by acid-dependent lysosomal proteases (15). The simplest interpretation is that to regulate seeding VCP processes cytoplasmic seeds acutely, based on extraction and proteasomal degradation of tau monomer (Fig. 9).

### VCP cofactors dictate the fate of tau seeds

Multiple efforts have attempted unsuccessfully to target VCP with small molecules to treat cancer (55). Our observations with ML-240 and NMS-873, which we originally predicted would have the same effect on tau seeding, highlighted the mechanistic complexity of this enzyme. Indeed, we found that specific cofactors were necessary for VCP to control seed amplification vs. destruction. Knockout of FAF2, also known as UBXD8, strongly enhanced tau seeding. It also caused spontaneous aggregation in the biosensors that we had never before observed. FAF2 was recently reported to facilitate VCP-dependent disaggregation of stress granules (56). It is a component of ERAD, with important roles also in lipid droplet biogenesis and maintenance (57,58). Whether its effects on tau seeding are due to direct recruitment and VCP activity on tau seeds or seed processing via one of its known pathways remains unclear. By contrast, knockdown of NPLOC4 increased tau levels yet inhibited seeding, consistent with a role in seed amplification. Genetic deletion of ATXN3 (Ataxin 3), a deubiquitinase, also suppressed tau seeding, but without changing tau levels. Ataxin 3 is proposed to facilitate ubiquitinated substrate release from VCP by cleaving ubiquitin chains to a minimum length for proteasomal degradation (59). However, its exact role in processing VCP substrates remains unclear. In the absence of its deubiquitinase, Cdc48 in yeast cannot thread its substrate through the core (60). Thus, ATXN3 KO might also prevent fragmentation of assemblies. Taken together, our results point to a complex molecular machine, likely with cell-specific components, that determines the fate of tau seeds.

### Multiple roles of VCP in degenerative disorders

Distinct VCP mutations cause VT (21) or MSP with ubiquitinated aggregates of TDP-43 (61,62). MSP mutants have been proposed to be hyperactive (63,64) whereas VT could result from VCP hypoactivity (21). However, *in vitro* experiments of ATP hydrolysis in isolation likely cannot account for the complexity of VCP mutations in a cellular setting. Indeed, others suggest that they might alter cofactor binding (59,64,65), perhaps explaining the diverse phenotypes. Multiple reports, including ours, now indicate that VCP regulates aggregation of TDP-43, a-synuclein, and tau through diverse mechanisms including autophagy, endolysosomal degradation, and disaggregation (21,66,22,20).

We must now consider VCP’s role early in the seeding process, and the specific cofactors involved in these activities. These could be conformation (strain) or amyloid-specific. Indeed, others identified UBXN6/UBXD1 as a VCP cofactor that regulates a-synuclein seeding in neurons (22). It is possible that neurons might differentially utilize cofactors to regulate tau seeding, and this will await additional comprehensive study. Ultimately, inhibition of specific sub-functions of VCP by modulating key cofactor interactions might be the only way to therapeutically target an enzyme otherwise critical for cell viability.

## Methods

### Cell Culture

HEK293T and U2OS cells were obtained from ATCC to create all cell lines. Cells were maintained in Dulbecco’s DMEM with 10% fetal bovine serum, 1% penicillin-streptomycin, and 1% GlutaMax. v2L tau biosensor cells were used for all seeding assays. Details on these biosensors have been recently published (8). Cell lines were checked approximately biweekly for mycoplasma contamination (Venor-GEM Mycoplasma Detection kit).

### Proteomics Screen

T225 flasks were coated with 10mL of 0.01mg/mL poly-D-lysine (PDL) for 3h in the incubator and rinsed with PBS before plating cells. 22 million cells were plated in 25ml/T225 flask and allowed to settle overnight. The following day, cells were treated with 50nM tau + Lipofectamine-2000 complexes (or 50nM α-synuclein + Lipofectamine as a negative control) which were incubated for 20min at RT prior to addition to cells. Cells were incubated with the fibrils for 5h. Thirty minutes before the 5h time point, cells were treated with BP (biotin phenol) at a final concentration of 500μM at 37°C. At 5h, cells were treated with H_2_O_2_ at a final concentration of 1mM and the flasks were agitated at RT for 1min. The biotinylation reaction was quenched with the quenching buffer followed by three additional rinses. Quenching buffer was also used to scrape the cells to collect the cell pellets. This buffer was prepared as previously described in the APEX2 labeling protocol (67).

### On-Bead Trypsin Digestion

Protein concentrations were normalized across all the samples (~1mg of starting lysate) based on the Pierce 660 assay readings and protein abundances from shotgun proteomics analysis of trypsin digests of these samples by the UT Southwestern Proteomics Core Facility. Lysates (1 mg) were incubated with 250μL of magnetic streptavidin beads at 4°C for overnight incubation ~16h. The next day, the beads were concentrated by magnet, and washed 2x with 200μL of 50mM Tris-HCl pH 7.5 followed by 2x with 2M urea + 50mM Tris-HCl pH 7.5. The beads were then incubated with 80μL 2M urea + 100μL 0.5ug/μl trypsin + 20μL 10mM DTT to achieve a final urea concentration of 1mM and a ratio of 1: 20 for trypsin: lysate, for 1h at 25°C with shaking at 1000rpm in a thermomixer. The beads were washed 2x with 60μl of 2M urea + 50mM Tris-HCl pH 7.5 and the two washes were combined with the supernatant. The eluate was reduced with DTT at a net concentration of 4mM by incubating for 30min with shaking at 1000rpm, 25°C. The samples were alkylated with 10mM iodoacetamide for 45min at 25°C with shaking at 1000rpm.

50mM Tris-HCl pH 7.5 was then added to achieve a final urea concentration of 0.73M. Samples were incubated overnight (~15h) at 37°C with shaking at 1000rpm to allow complete trypsin digestion. The samples were removed from the thermomixer and spun down. Trypsin was quenched by acidifying the samples to pH <3 with the addition of formic acid at a final concentration of 1%.

### TMT Mass Spectrometry

5μL of 10% trifluoroacetic acid (TFA) was added to each sample, and solid-phase extraction was performed on each sample using an Oasis HLB 96-well uElution plate (Waters). Eluates were dried and reconstituted in 50μL of 100 mM triethylammonium bicarbonate (TEAB). 10μL of each sample was labeled with 4μL of a different TMT10plex reagent (Thermo Scientific, label TMT10–131 not used). Samples were quenched with 1μL of hydroxylamine, mixed, and dried in a SpeedVac. Samples were reconstituted in 2% acetonitrile, 0.1% formic acid to a concentration of 0.5 ug/μL,

2μL of each TMT sample were injected onto an Orbitrap Fusion Lumos mass spectrometer coupled to an Ultimate 3000 RSLC-Nano liquid chromatography system. Samples were injected onto a 75μm i.d., 75-cm long EasySpray column (Thermo) and eluted with a gradient from 0–28% buffer B over 180 min. Buffer A contained 2% (v/v) acetonitrile (ACN) and 0.1% formic acid in water, and buffer B contained 80% (v/v) ACN, 10% (v/v) trifluoroethanol, and 0.1% formic acid in water. The mass spectrometer was operated in positive ion mode with a source voltage of 1.8 kV and an ion transfer tube temperature of 275°C. MS scans were acquired at 120,000 resolution in the Orbitrap and top speed mode was used for SPS-MS3 analysis with a cycle time of 2.5 s. MS2 was performed with CID with a collision energy of 35%. The top 10 fragments were selected for MS3 fragmentation using HCD, with a collision energy of 55%. Dynamic exclusion was set for 25 s after an ion was selected for fragmentation.

### Proteomics Data Analysis

Raw MS data files were analyzed using Proteome Discoverer v2.4 (Thermo), with peptide identification performed using Sequest HT searching against the human protein database from UniProt. Fragment and precursor tolerances of 10 ppm and 0.6 Da were specified, and three missed cleavages were allowed. Carbamidomethylation of Cys and TMT labeling of N-termini and Lys sidechains were set as a fixed modification, with oxidation of Met set as a variable modification. The false-discovery rate (FDR) cutoff was 1% for all peptides.

For every biological replicate, absolute abundance of each protein was first normalized to the total protein abundance of a particular lysate sample to account for any differences in total protein concentrations across samples before comparison. These values were used to calculate the relative enrichment of proteins specific to tau seeding: Protein Abundance Ratio= (sAPEX2 P301S + seeds)/(sAPEX2 P301S – seeds).

Normalized protein abundance ratios for sAPEX2 P301S and sAPEX2 alone (negative control) treated with and without tau fibrils were compared using unpaired t- test on three independent biological replicates; two-stage step-up (Benjamini, Krieger, and Yekutieli), FDR 1.00%). Positive relative abundance values on the graph indicate enrichment in the aggregation proteome. Statistical significance was determined based on q value.

### Generation of TDP-43 Biosensor Cell Line

FM5 CMV promoter plasmids containing the mClover3 and mRuby3 fluorophores were digested with Esp3I restriction enzyme. WT TDP-43 (a.a. 274–414) was cloned into the cut plasmids using Gibson assembly to generate constructs expressing TDP-43 tagged to the respective fluorophores. Constructs were sequence verified using UTSW’s Sanger sequencing core and used for making lentivirus.

HEK293T cells were transduced with the TDP-43 lentivirus and were sorted into monoclonal cells for highest expression of the two fluorophores. Monoclonal cell line that responded with maximum seeding to different FTLD TDP-43 lysates was chosen for the seeding assay reported here. Cells expressing the individual fluorophores were also sorted as fluorophore compensation controls for detecting FRET on the flow cytometer.

### Biosensor Seeding Assay

All biosensor assays were performed with “naked seeding” (no cation-based transfection reagent). Cell lines were plated at a density of 15,000 cells/well of a 96 well plate and allowed to settle overnight. Cells were treated with an appropriate concentration of recombinant tau fibrils for 48h, and then harvested for flow cytometry. Fibril preps were sonicated in a water bath sonicator for 1min at 65Amp prior to cell treatment. Recombinant tau fibrils were prepared as previously described (30).

For seeding with brain lysates and for the a-synuclein biosensor seeding assay, 8,000 cells/well (10,000 cells/well for TDP-43 biosensors) were plated in 96 well plates and seeding was monitored for 72h. In the case of brain homogenates, biosensors were treated with 25μg of tauopathy lysates and 10μg of FTLD lysate. These were sonicated for 1min at 65 Amp in a water bath sonicator. For seeding with a-synuclein, fibrils were sonicated for 5min total, 1min on/1min off at 65 Amp, and used at a final concentration of 400nM.

All seeding results are reported as % FRET+ cells. The FRET data were plotted by subtracting the background signal (no exogenous tau added) which was negligible for all conditions (no FRET recorded in the absence of tau seeds), unless otherwise specified.

### Brain Homogenization

Brain tissue from clinically and neuropathologically characterized cases of AD, CBD, and FTLD TDP-43 were obtained from UTSW and Washington University in St. Louis. All human tissues used in these experiments were derived from autopsy subjects. Since deceased subjects are not considered human subjects for research purposes, these studies were exempt from human subjects research regulations and did not require IRB approval. Brain samples were weighed and added to 1X TBS supplemented with cOmplete Ultra (Roche) protease inhibitor to prepare a 10% w/v solution. The brains were homogenized using a probe homogenizer to obtain a slurry that was sonicated for 15min total, 1min on/30sec off. The sonicated samples were centrifuged at 4°C for 15min at 21,300 × g. Protein concentration of the supernatant was measured using Pierce 660 assay and was subsequently used for naked seeding.

### Uptake Assay

The uptake assay was performed as previously described (30). v2L cells were plated overnight at a density of 8,000 cells/well of a 96 well plate. Cells were treated with 25nM of AF-647 labeled tau fibrils or AF-647 dye alone as a negative control. After 4h of incubation with the fibrils, cells were harvested with 0.25% trypsin for flow cytometry.

The labeled fibrils used in this assay were obtained by incubating recombinant tau fibrils (8μM, 200μL) with lyophilized AF-647 dye (25μg) for 1h at room temperature (RT) followed by quenching the reaction with 100mM glycine and subsequent dialysis in a 3500kDa dialysis cassette.

The median fluorescence intensity (MFI) values representing the amount of tau internalized were plotted after subtracting the background MFI of the dye alone signal for all conditions. These MFI values were then normalized relative to the appropriate control condition of the respective experiment (DMSO, NTG, or Scr controls).

### Compound Treatments

96 well plates were coated with 0.01mg/mL PDL and incubated at 37°C for 3h followed by washout with PBS. v2L cells were plated at a density of 15,000 cells/well and allowed to settle overnight. Cells were treated with different compounds (ML-240, NMS-873, MG132, and LLOMe) for about an hour upon which 25nM of recombinant tau fibrils were introduced to the media. After four hours of incubation with the fibrils (five hours with compounds), the media was replaced with fresh media and uptake or seeding was monitored for 4h and 48h, respectively.

### Flow Cytometry

To harvest cells for flow cytometry, media was removed, and cells were treated with 0.05% trypsin (0.25% trypsin for uptake assay) for 5min at 37°C (0.25% trypsin, 15min at 37°C in case of PDL coated plates). Trypsin was quenched with cold media and cells were resuspended a few times before transferring the suspension to 96-well round-bottom plates which were centrifuged at 1000 rpm for 5min. Supernatant was removed and the cell pellets were resuspended in 2% paraformaldehyde (PFA) and allowed to fix for 10min at RT. Cells were spun down again, PFA was removed, and cells were resuspended in PBS and stored at 4°C until ready to be run on a flow cytometer (BD LSRFortessa) for quantifying the FRET signal.

### Cloning

FM5 vector with UBC promoter was used to clone all the APEX constructs. sAPEX fragments (AP and EX) were PCR amplified from the constructs provided by the laboratory of Alice Ting (Stanford). Amplified sAPEX fragments were appended on to the c-terminus of RD tau fragments via a linker using overlap PCR. Using Gibson assembly, the final gene fragments were cloned into FM5 UBC plasmid, which was cut with Esp3I. All Gibson reaction products were transformed into Stbl3 bacterial cells. Bacterial colonies were inoculated, DNA was purified using Qiagen miniprep kit, and the sequences were verified using Sanger sequencing at UTSW’s sequencing facility.

### Lentivirus Production

Low passage HEK293T cells were plated at ~ 70% confluency in 6 well plates and allowed to settle overnight. A master mix was prepared using 400ng of plasmid of interest, 400ng of VSVG, and 1200ng of PSP plasmids required for virus packaging, along with 7.5mL of TransIT 293T and 120ul of OMEM per well of a 6 well plate. The master mix was allowed to incubate at RT for 30min upon which it was added to the cells drop-wise. The virus was harvested 48h later by collecting the media, spinning it for 5min at 1000rpm, and then freezing the aliquoted supernatant.

### Differentiation and Culturing of Human iPSC-derived Cortical Neurons

We utilized the integrated, inducible, and isogenic Ngn2 iPSC line (i^3^N). It was previously shown that expression of the transcription factor neurogenin-2 (Ngn2) induces rapid differentiation of iPSCs into cortical glutamatergic neurons (68). This iPSC line harbors a doxycycline-inducible mouse Ngn2 transgene at an adeno-associated virus integration site 1 (AAVS1) safe-harbor locus, allowing for a simplified differentiation protocol (69,70). iPSCs were dissociated with Accutase (Sigma, A6964) and plated onto basement membrane extract-coated plates (R&D, 3434–001-02). Ngn2 expression was induced with 2 μM doxycycline hyclate (Sigma, D9891) in KSR media alone with 10 μM SB431442 (R&D, 1614), 2 μM XAV939 (Stemgent, 04–0046) and 100 nM LDN-193189 (Stemgent, 04–0074) (doxycycline hyclate is maintained in all medias going forward). On day 2, cells were fed with a 1:1 ratio of KSR media + SB/XAV/LDN and N2-supplmented neural induction media with 2 μg/ml puromycin (Life Technologies, A1113803). On day 3, cells were fed with N2-supplemented neural induction media. On day 4, cells were dissociated with Accutase and plated onto PDL-(Sigma, P1149) and laminin-(Life Technologies, 23 017–015) coated tissue culture plates. Cells were subsequently maintained with neurobasal media (Life Technologies, 21103049) supplemented with NeuroCult SM1 (StemCell Technologies, 05711) and 10 ng/ml brain-derived neurotrophic factor (R&D, 248-BD-005/CF) until collected.

### Compound Treatment and Seeding Assay in Neurons

Differentiated human neurons were treated with tau RD P301S lentivirus (tau-clover at an MOI of 3; tauruby at an MOI of 2). 48h later, the transduced cells were simultaneously incubated with 15nM FL, WT recombinant tau fibrils and the VCP inhibitors, ML-240 and NMS-873 at 100nM for 48h. For acute exposure with tau fibrils and the compounds, the neuronal biosensors were treated with 1μM ML-240 and 100nM NMS-873 for 4h followed by media replacement. FRET signal, used as a measure of tau aggregation was monitored over 48h using ImageXpress Confocal HT.ai High-Content Imaging System (Molecular Devices). 4 images per well, with 5 wells per condition of a 96 well plate, were collected for the clover, ruby, and FRET channels. Single color controls were used to correct for fluorophore bleed through using an automated algorithm on MATLAB. Single color controls were used to measure the bleedthrough coefficient of each channel by fitting the correlation of pixel intensities in the single color channel and the “empty” channel with a linear fit. Images from the acceptor/donor channels of real data were then multiplied by the resulting slopes (i.e. bleedthrough coefficients) to approximate the amount of bleedthrough. Finally, FRET channel images were corrected by subtracting these bleedthrough images from the FRET images. The corrected images were analyzed to calculate the FRET area using an ImageJ macro and the quantified FRET area was plotted for different conditions.

### CRISPR/Cas9 Screen for VCP Cofactors

CRISPR constructs for the cofactors were outsourced to Twist Biosciences for synthesis. Constructs not synthesized by the company were cloned in the lab using standard ligation reactions. Four guides per gene were chosen from the Brunello library deposited online and ordered as duplex DNA from IDT. LentiCRISPRv2 (Addgene #52961) was cut using Esp3I, and T4 ligase was used for all ligation reactions of the guides into the plasmid. Stbl3 bacteria were transformed with the ligated products, selected colonies were inoculated, mini-prepped using Qiagen kit, and the purified DNA was sequence-verified. Pooled lentivirus was prepared with four constructs per gene and v2L cells were transduced with the virus at the desired MOI. After 24h, cells were expanded in puromycin media (2mg/mL) for selection of the KO population. Selected populations were eventually used for seeding and uptake assays. The KO was verified using western blot.

### siRNA Knockdown

siRNAs were ordered from Origene. 300,000 v2L cells were plated in 6 well plates and allowed to settle overnight. The next day, cells were treated with 100nM of each siRNA, with a total of three siRNAs per gene using RNAiMax Lipofectamine (Thermo) as a transfection vehicle at 7.5μl/well. After 48h of transfection, the cells were plated in 96 well plates for seeding and uptake assays. Cells were also used for western blot to verify the knockdown.

### Western Blot

Cell pellets were lysed in RIPA buffer and allowed to sit on ice for 5min followed by a 15,000g spin for 10min at 4°C. The supernatants were used to determine the protein concentrations using Pierce 660 assay. 15mg of total protein was treated with SDS buffer and heated at 95°C for 10min. Samples were loaded onto 4–12% bis-tris gels and the proteins were transferred onto nitrocellulose membranes using the Biorad turbo transfer machine. All incubations for subsequent steps were done in TBS + 0.05% Tween-20 (TBST). The membranes were first incubated in blocking buffer (5% milk powder + TBST) for 1h at RT, followed by primary antibodies in the blocking buffer at 4°C with overnight shaking. After the primary antibody incubation, the membranes were washed 3x with TBST, 10min each. Then, appropriate HRP-conjugated secondary antibodies in blocking buffer were added to the membranes for a 1.5h incubation at RT. Membranes were again washed 3x in TBST followed by a single wash in TBS alone before reading the HRP signal using the Thermo ECL kit.

### Statistical Analysis

Proteomics data was analyzed using unpaired t- test, two-stage step-up (Benjamini, Krieger, and Yekutieli), FDR 1.00%. One-Way ANOVA (Šídák method) with a 95% confidence interval was used for all the other statistical analyses unless otherwise stated. Paired t-test was used to analyze the neuron data.

The P values are described as follows: ns = not significant/P>0.05, * = P ≤ 0.05, ** = P ≤ 0.01, *** = P ≤ 0.0001, *** = P ≤ 0.0001

### Graphics

Biorender.com was used to create the graphics presented here and has granted publication and licensing rights (agreement number: RX26PLQVWQ).

## Figures and Tables

**Table 1:** List of VCP cofactors and their proposed functions

Cofactor	Function
AMFR/GP78	ERAD
AMKZF1	Cellular response to hydrogen peroxide, ERAD
ASPSCR1/ UBXD9	VCP hexamer disassembly
ATXN3	Deubiquitinase; ERAD
DERL1	ERAD
DERL2	ERAD
FAF1/ UBXD12	Apoptosis, autophagy
FAF2/ UBXD8	ERAD, lipid droplet turnover
NGLY1	Degradation of misfolded glycoproteins
NPLOC4/ NPL4	ERAD
NSFL1C/ p47	Membrane fusion
OTUB1	Cleaves branched polyubiquitin chains
PLAA	ERAD, autophagy
RPS27A	Fusion of ubiquitin and ribosomal protein S27a
SVIP	ERAD, autophagy
SYVN1	E3 ligase; ERAD
UBE4B	E3/E4 ligase; ERAD
UBXIM1/SAKS1	ERAD
UBXN10/UBXD3	Tethering factor for VCP in cilium assembly
UBXN11/ UBXD5	Actin cytoskeleton reorganization
UBXN2A/ UBXD4	Autophaqosome formation, proteasome degradation
UBXN2B/ p37	Membrane fusion
UBXN4/ UBXD2	ERAD
UBXIN6/UBXD1	ERAD, endosome to lysosome transport, macroautophagy
UBXN7/ UBXD7	HIF1a turnover
UBXN8/ UBXD6	ERAD
UFD1L	ERAD
VCPIP1	Deubiquitinase, membrane fusion
VIMP	ERAD
YOD1	Deubiquitinase, macroautophagy, ERAD

## Data Availability

Data generated in this study but not presented here are available from the corresponding author on request.

## List Of Abbreviations

VCP/p97: valosin containing protein
sAPEX2: split ascorbate peroxidase 2
RD: repeat domain
FL: full length
WT: wild type
BP: biotin-phenol
TMT-MS: tandem mass-tag mass spectrometry
KO: knockout
KD: knockdown
AF-647: Alexa fluor-647
AD: Alzheimer’s disease
CBD: corticobasal degeneration
TDP-43: TAR DNA-binding protein 43
